# Assessment of the Association between Entropy in PET/CT and Response to Anti-PD-1/PD-L1 Monotherapy in Stage III or IV NSCLC

**DOI:** 10.3390/life13041051

**Published:** 2023-04-20

**Authors:** Julie Malet, Julien Ancel, Abdenasser Moubtakir, Dimitri Papathanassiou, Gaëtan Deslée, Maxime Dewolf

**Affiliations:** 1 Department of Respiratory Diseases, Reims University Hospital, 45, Rue Cognacq-Jay, 51100 Reims, France; 2 INSERM UMRS 1250, University of Reims Champagne-Ardenne, 51100 Reims, France; 3 Department of Nuclear Medicine, Institut Godinot, 1, Rue du Général Koenig, 51100 Reims, France; 4 UFR de Médecine, Reims-Champagne Ardenne University, 1, Rue Cognacq-Jay, CEDEX 51095 Reims, France; 5 CReSTIC (Centre de Recherche en Sciences et Technologies de l’Information et de la Communication), EA 3804, University of Reims Champagne-Ardenne, Moulin de la Housse, BP 1039, CEDEX 51687 Reims, France

**Keywords:** entropy, immune checkpoint inhibitors, non-small cell lung cancer, positron-emission tomography

## Abstract

Anti-PD-1/PD-L1 therapy indications are broadened in non-small cell lung cancer (NSCLC) although immune checkpoint inhibitors (ICI) do not provide benefits for the entire population. Texture features based on positron emission tomography/computed tomography (PET/CT), especially entropy (based on a gray-level co-occurrence matrix (GLCM)), could be interesting as predictors in NSCLC. The aim of our retrospective study was to evaluate the association between GLCM-entropy and response to anti-PD-1/PD-L1 monotherapy at the first evaluation in stage III or IV NSCLC, comparing patients with progressive disease (PD) and non-progressive disease (non-PD). In total, 47 patients were included. Response Evaluation Criteria in Solid Tumors (RECIST 1.1) were used to evaluate the response to ICI treatment (nivolumab, pembrolizumab, or atezolizumab). At the first evaluation, 25 patients were PD and 22 were non-PD. GLCM-entropy was not predictive of response at the first evaluation. Furthermore, GLCM-entropy was not associated with progression-free survival (PFS) (*p* = 0.393) or overall survival (OS) (*p* = 0.220). Finally, GLCM-entropy measured in PET/CT performed before ICI initiation in stage III or IV NSCLC was not predictive of response at the first evaluation. However, this study demonstrates the feasibility of using texture parameters in routine clinical practice. The interest of measuring PET/CT texture parameters in NSCLC remains to be evaluated in larger prospective studies.

## 1. Introduction

Lung cancer is the leading cause of cancer-related death worldwide. Its incidence declined by almost 3% annually in men and 1% annually in women from 2009 to 2018 [1]. The five-year relative survival rate for lung cancer is 22%. Non-small cell lung cancer (NSCLC) represents about 80% of all lung cancer cases. Major improvements have been made in NSCLC treatment with the advent of targeted therapies and immune checkpoint inhibitors (ICIs), specifically programmed cell death protein 1 (PD-1) and its ligand (PD-L1) inhibitors [2]. ICIs require the reactivation of a pre-existing immune response and its efficacy is related to the presence of necrosis, hypoxia, inflammation, and immune effector cells in the tumor sites [3].

However, immunotherapy does not provide benefits for the entire population and the identification of biomarkers and prediction tools remains necessary to select the ideal candidate population. Nowadays, the only approved biomarker for immunotherapy is PD-L1 status, which is not ideal because of tumor heterogeneity of expression and dynamic changes in PD-L1 expression [4]. 

In this context, emerging image analysis techniques, such as radiomics, seem to be interesting and have the advantage of being non-invasive. Based on standard-of-care, these are widely available and sample the entire tumor. They refer to the comprehensive quantification of tumor phenotypes by applying a large number of quantitative image features. They provide distinct information from laboratory tests, and genomic or proteomic assays [5]. These features, combined with other information, can be correlated with clinical outcome data and used for evidence-based clinical decision support [6]. Moreover, radiomics can be used as a prognosis tool, especially in lung cancer [7]. Artificial intelligence can be combined with radiomics to improve both tumor characterization, such as for certain tumor molecular traits, the relationship with tumor dissemination, and prognosis [8].

This contrasts with the traditional practice of treating medical images as pictures intended solely for visual interpretation, where tumor response is only measured using one- or two-dimensional descriptors of tumor size (RECIST and WHO, respectively). Although a change in tumor size can indicate response to therapy, it often does not predict overall or progression-free survival [9]. Indeed, computed tomography (CT) texture analyses allow one to evaluate the distribution and relationship of pixel or voxel levels. In a statistical-based model, first-order statistics evaluate the gray-level frequency distribution from the pixel/voxel intensity histogram in each area of interest, regardless of the spatial relationships, including, for example, mean intensity, energy, entropy, skewness, and kurtosis. Second-order statistics are textural features quantifying tumor heterogeneity by analyzing the spatial distribution of pixel/voxel intensities. These are based on a gray-level co-occurrence matrix (GLCM), including second-order entropy, homogeneity, contrast, energy, dissimilarity, and correlation [10]. GLCM-entropy has been associated with worse prognosis in some studies [11,12], particularly in NSCLC patients receiving nivolumab [3]. Third-order statistics such as short run emphasis (SRE) are based on a gray-level run-length matrix (GLRLM). Here, a run length is considered to be a number of neighboring pixels that possess the same gray intensity in a particular direction.

Texture analysis can be applied in [18F]-fluoro-2-deoxy-d-glucose (FDG) positron emission tomography (PET)/CT and was first reported in 2009 by El Naqa et al. for patients with cervix and head and neck cancers [13]. Texture parameters have been shown to have predictive value in various types of cancer [14,15].

Prior studies applied radiomics in PET/CT in NSCLC receiving checkpoint blockade immunotherapy and showed that a PET/CT-based signature can be used prior to the initiation of immunotherapy as a predictive biomarker for response [16]. In particular, among radiomics, entropy, which measures texture irregularity in terms of the randomness of the gray-level distribution inside the region of interest, has a good test–retest repeatability, suggesting a good reliability [17]. Entropy seems to be interesting in terms of discerning responder (overall survival (OS) > 14.9 months) from non-responder patients receiving pembrolizumab in stage IV NSCLC [18]. In stage III NSCLC, it seems to be a prognostic factor of two-year progression-free survival (PFS) [19].

The primary aim of our retrospective study was to evaluate the association between GLCM-entropy in 18F-FDG PET/CT and response to anti-PD-1/PD-L1 monotherapy at first evaluation in extensive-stage NSCLC, comparing patients with progressive disease (PD) and non-progressive disease (non-PD). Secondary endpoints were used to evaluate the prognostic value of GLCM-entropy for OS and PFS, the correlation between GLCM-entropy and PD-L1 tumor proportion score (TPS) status, metabolic tumor volume (MTV), standard uptake value (SUV), total lesion glycolysis (TLG), and GLRLM-short run emphasis (SRE) measured using PET/CT.

## 2. Materials and Methods

### 2.1. Population

**Figure 1:**
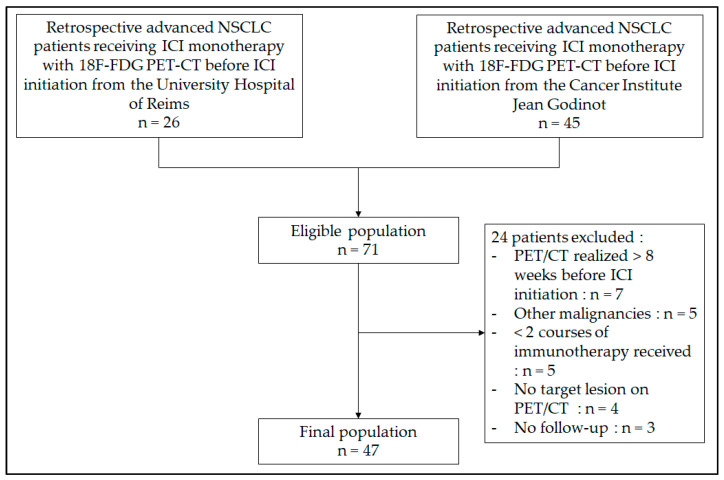
Flow chart.

In total, 47 patients matching the following inclusion criteria were enrolled from September 2015 to February 2021 at the Cancer Institute Jean Godinot and the University Hospital of Reims (France): age > 18 years old, histologically confirmed stage III NSCLC (not eligible for local treatment) or stage IV NSCLC (8th TNM classification of the international Association for the Study of Lung Cancer), no other malignancies, receiving anti-PD-1/PD-L1 monotherapy (atezolizumab, nivolumab, or pembrolizumab), with a 18F-FDG PET/CT realized at the Cancer Institute Jean Godinot in the 8 weeks before immunotherapy initiation, and who had received a minimum of 2 courses of immunotherapy (Figure 1).

The exclusion criteria were 18F-FDG PET/CT realized outside of the Cancer Institute Jean Godinot, other associated malignancies, a histology other than NSCLC, chemotherapy associated with immunotherapy, any other treatment received between 18F-FDG PET/CT and immunotherapy initiation, and no target lesion visible in the 18F-FDG PET/CT.

### 2.2. Study Protocol

In this retrospective observational study approved by the Cancer Institute Jean Godinot and the University Hospital of Reims’ data protection departments (MR00424012022), all patients receiving ICIs had 18F-FDG PET/CT before the initiation of ICIs and CT or PET/CT at first follow-up. 

The baseline clinical characteristics (age, sex, Eastern Cooperative Oncology Group (ECOG) scale, histology, TNM stage in pre-therapeutic PET/CT, and smoking status) and PD-L1 TPS status, 18F-FDG PET/CT date, first course date, line of treatment, molecule of ICI received, date of first follow-up, and date of death or the last contact were obtained from the medical records.

Response Evaluation Criteria in Solid Tumors (RECIST 1.1) were used to evaluate response to immunotherapy [20]. Complete response (CR) was the disappearance of all target lesions, partial response (PR) was a decrease of 30% or more in the sum of the diameters of target lesions, progressive disease (PD) was an increase of 20% or more in the sum of the diameters of target lesions, and stable disease (SD) was noted in patients whose tumors did not show either sufficient shrinkage to qualify for PR nor sufficient increase to qualify for PD. When a CT evaluation was not available due to fast disease progression or the worsening of the patient’s clinical condition, the response was assessed by a clinical and laboratory evaluation. Patients with PD were opposed to patients with non-progressive disease including CR, PR, and SD patients.

### 2.3. Image Acquisition and Analysis

18F-FDG PET/CT acquisitions were performed with a standard protocol following the recommendations of the European Association of Nuclear Medicine [21]. Patients were instructed to fast for at least 6h before the scan, and a blood glucose test was performed before the injection of 18F-FDG (<200 mg/dl). They received an injection of 3 MBq/kg of 18F-FDG followed by a 60 min uptake period. 18F-FDG PET acquisition was made from the root of the thighs to the vertex, 2 to 2.5 min per bed position (11% overlap), via tomography, i.e., General Electric Healthcare Discovery ^®^ 710 model that was put into service in 09/2015, with the following parameters: reconstruction using fully 3D TOF with a sharp IR point-spread function reconstruction algorithm, 2 iterations per 24 sub-sets, a matrix size of 256 × 256, a 6.7 mm slice thickness, and a 2.73 × 2.73 × 3.25 mm^3^ voxel size. All PET/CT images were analyzed on a dedicated workstation and independently interpreted by a nuclear medicine physician who was aware of the clinical data. A RECIST assessment was performed by two pneumologists and was reviewed by a third in case of disagreement. They were blinded to the outcome of the study.

### 2.4. Radiomic Feature Extraction

A total of six radiomic features were extracted from primary lung tumors, including four volume-based features (SUVmax, SUVmean, MTV, and TLG), one texture-based heterogeneity feature derived from the gray-level co-occurrence matrix (entropy), and one texture-based heterogeneity feature derived from the gray-level run-length matrix (SRE) using the open-access LIFEx platform v4.0 (IMIV/CEA, Orsay, France).

Primary lung tumors were semi-automatically segmented, and regions of interest were further reviewed and corrected by a physician that was blinded to the outcome label. When there was no pulmonary lesion, features were extracted from the extra-pulmonary target lesion with the highest SUVmax.

### 2.5. Statistical Analyses

Data are presented as median and range or effective and percentages for quantitative and qualitative variables, respectively; the red bar in the scattergrams represents the median. The associations between features were studied using the Fisher test. The Kolmogorov test showed that distributions were not normal. Quantitative data were analyzed using non-parametric tests (Mann–Whitney or Kruskal–Wallis as appropriate) to assess the significance between different conditions. A Spearman test was used to study the correlation. Non-supervised clustering was performed with the decision tree CHAID method, with a Bonferroni correction. The Kaplan–Meier method was used to perform the survival analysis. In all exploratory analyses, results with a two-sided *p*-value ≤ 0.05 were considered significant. The XLSTAT software (version 2022.1, Addinsoft company, Paris, France) was used to analyze and reformat the data.

## 3. Results

### 3.1. Population Characteristics

In total, 47 patients were enrolled: 14 females and 33 males, with a median age of 64 years (range: 32–84 years old). Six out of forty-seven patients were non-smokers, forty out of forty-seven were current of former smokers. In total, 63% (30/47) of patients had adenocarcinoma, while 31.9% (15/47) had squamous cell carcinoma. PD-L1 TPS status was available for 33 out of 47 patients (70%). Twenty-five patients received nivolumab, twenty received pembrolizumab, and two received atezolizumab. Immunotherapy was the first line therapy for 31.9% of patients (15/47) and the second line therapy for 40.4% of patients (19/47). A full list of patient characteristics is presented in Table 1.

### 3.2. PET/CT, Follow-Up, and Response to Immunotherapy

The median time from PET/CT to the start of immunotherapy was 16 days (range 0–48 days). The median time to first evaluation was 76 days (range 41–205; IQR 60–90 days). At the first evaluation, 25 patients were PD, 14 were SD, 6 were PR, and 2 were CR. In two cases, CT evaluation was not available and disease progression was observed by clinical evaluation.

In first line of treatment, five patients (33.3%) were PD. In second line of treatment or subsequent treatments, 20 (62.5%) patients were PD without significant differences (*p* = 0.060). Despite a trend, PFS did not significantly differ between patients treated in the first line (*p* = 0.074). As expected, OS was significantly better for patients treated in the first line in comparison with patients in the second line or subsequent treatments, with a respective median not being reached vs. 9.0 months (*p* = 0.023).

At the last evaluation, 34% patients (16/47) were alive and 66% were dead (31/47). ICI treatment was ongoing at the time of the last follow-up in 4/47 patients. For others, the causes of ICI suspension were disease progression in 35 cases, death in 4 cases, complete response in 1 case, and other reasons in 2 cases (pneumonia and patient’s choice).

### 3.3. Radiomics Analysis

**Figure 2:**
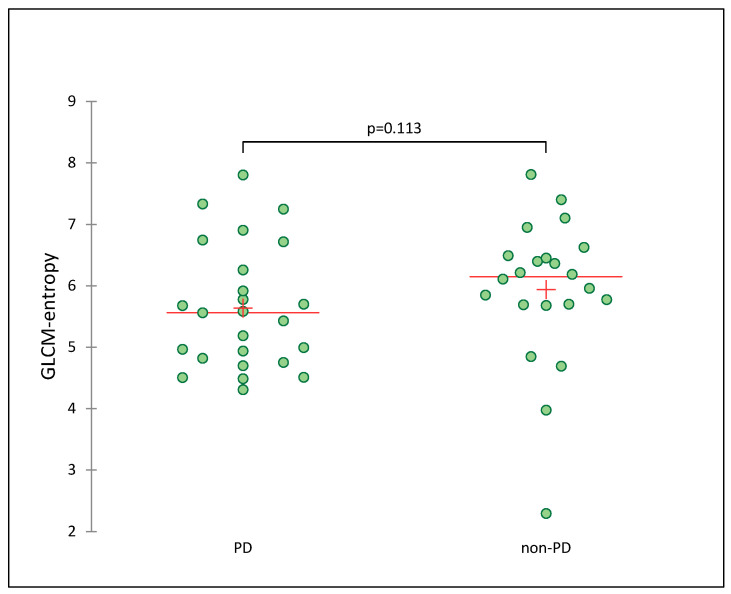
Scattergram showing the association between GLCM-entropy and response to anti-PD-1/PD-L1 monotherapy at first evaluation.

**Figure 3:**
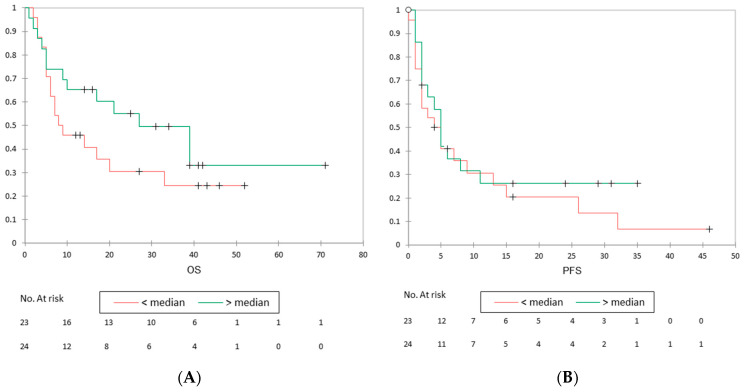
Association between GLCM-entropy and OS and PFS. (**A**). Kaplan–Meier plot showing prognostic value of GLCM-entropy for the overall survival (OS) (*p* = 0.220). (**B**) Kaplan–Meier plot showing prognostic value of GLCM-entropy for the progression-free survival (PFS) (*p* = 0.393). Crosses represent censored patients.

The values of PET/CT parameters are listed in Table 2. The median value for GLCM-entropy was 5.78. GLCM-entropy tended to show an association between patients with progressive disease at first follow-up (5.56) compared to patients with non-progressive disease (6.15), but this was not statistically significant (*p* = 0.113, Table 3 and Figure 2). The prognostic value of GLCM-entropy for PFS and OS is shown in Figure 3. GLCM-entropy was neither associated with PFS (*p* = 0.393) nor OS (*p* = 0.220). In the sub-group analyses, the impact of GLCM-entropy on OS did not vary according to histology sub-type (squamous vs. non-squamous NSCLC, *p* = 0.344 and *p* = 0.067, respectively). Moreover, the prognostic value of GLCM-entropy for OS was not associated with PD-L1 TPS status (*p* = 0.791 among PD-L1 < 50% and *p* = 0.168 among PD-L1 ≥ 50%). Whether anti-PD-1/PD-L1 monotherapy was administered in the first, in the second, or in the subsequent line, GLCM-entropy was not predictive of OS.

Furthermore, median SUVmax values tended to be different in the two groups (PD vs. non-PD patients, *p* = 0.103). No significant association was found for median SUVmean values (*p* = 0.519), for the other conventional parameters (TLG and MTV), or for GLRLM-SRE.

To ensure that no confounding factors affected the prognosis between the two groups, we controlled and did not observe any association between GLCM-entropy and patient characteristics (Table 4).

As observed in the global population, PFS did not differ according to GLCM entropy sub-groups for patients in the first line (*p* = 0.926) or in the second line or subsequent lines of treatment (*p* = 0.303). Regarding OS, GLCM-entropy was not associated with a better clinical outcome (*p* = 0.467). However, a trend was noticed for patients in the second line or subsequent lines: patients with higher GLCM-entropy exhibited a better median OS than patients with a GLCM-entropy below the median (7.3 vs. 26.3 months, respectively, *p* = 0.068) (Supplementary Figure S1).

**Figure 4:**
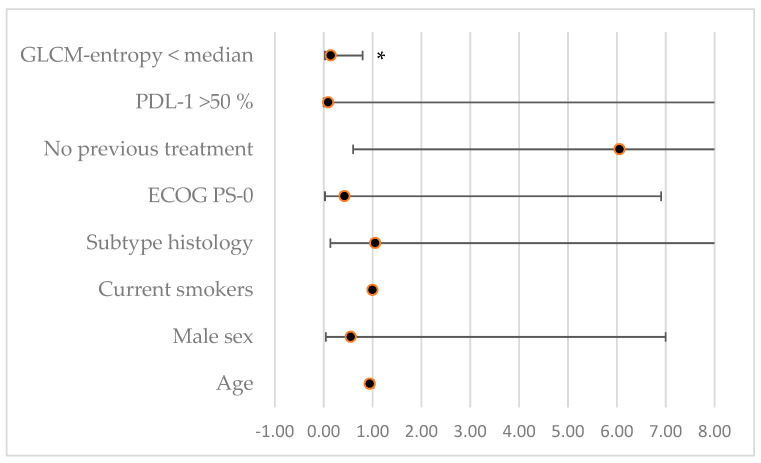
Forest plot illustrating associations (hazard ratio) between progressive disease status for patients treated with immunotherapy and various parameters in multi-variate analysis; * denotes a significant association, *p* < 0.05.

Finally, we performed a multi-variate analysis to limit potential confounding factors between the explored parameters (Table 5, Figure 4). Interestingly, only GLCM-entropy remained significantly associated with PD (HR = 0.14, *p* = 0.03). A trend was also noticed for others factors usually associated with worse prognosis, such as age and line of treatment.

**Figure 5:**
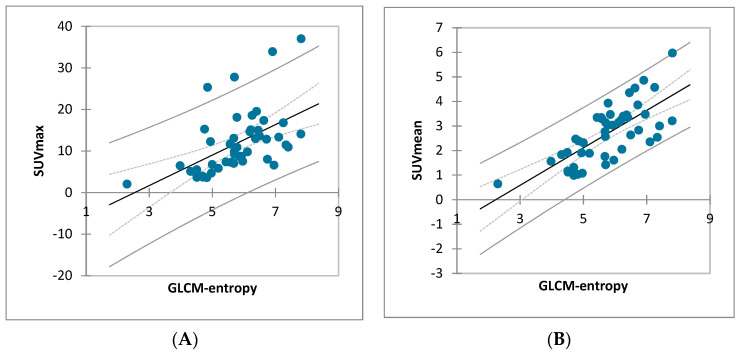
Correlation between GLCM-entropy and standard uptake value (SUVmax and SUVmean). (**A**) SUVmax linear regression by GLCM-entropy (r = 0.532, R^2^ = 0.283), *p* < 0.0001 (95 IC: 0.278–0.786). (**B**) SUVmean linear regression by GLCM-entropy (r = 0.725, R^2^ = 0.525), *p* < 0.0001 (95 IC: 0.518–0.932).

**Figure 6:**
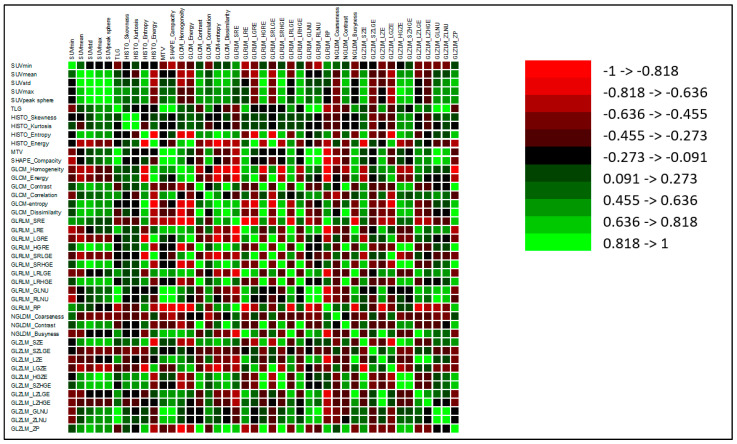
Correlation matrix. Green cases represent positive correlation and red cases display negative correlation with the relevance of the correlation according to the intensity scale. Coefficient of regression for each association is reported in Supplementary Table S1.

No correlation was found between GLCM-entropy and MTV (*p* = 0.485) or TLG (*p* = 0.807), but a significant correlation was found between SUVmax (r = 0.532, *p* < 0.0001 (95 IC: 0.278–0.786)) and SUVmean (r = 0.725, *p* < 0.0001 (95 IC: 0.518–0.932)) (Figure 5). All these results are shown in the correlation matrix (Figure 6).

**Figure 7:**
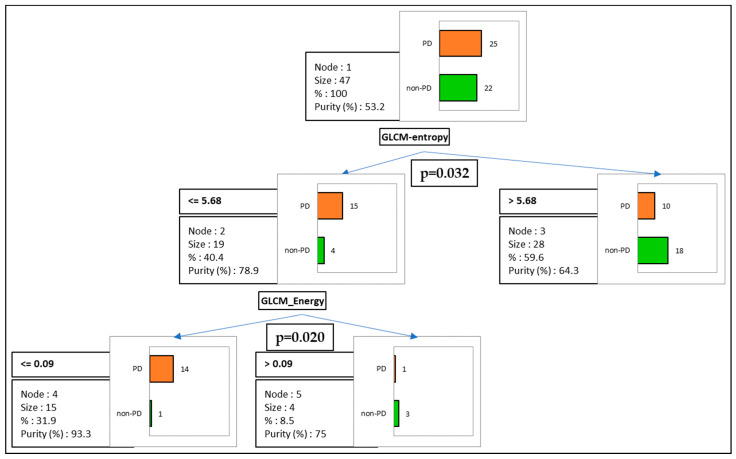
Unsupervised clustering between all texture parameters, classified into a predictive model of patients with progressive disease (PD; orange) vs. non-progressive disease (non-PD; green). The first node is represented by GLCM_Entropy (*p* = 0.032), followed by GLCM_Energy (*p* = 0.020).

Finally, we performed unsupervised clustering based on CHAID method, analyzing all texture parameters and exploring their respective potential to predict PD vs. non-PD patients (Figure 7). Strikingly, GLCM_Entropy remained the main factor that was able to discriminate PD from non-PD patients (*p* = 0.032). According to the GLCM_Entropy node, 18 non-PD patients (81%) from 22 in total were accurately identified, highlighting the relevance of GLCM_Entropy in predicting the clinical outcome in NSCLC patients treated with immunotherapy.

## 4. Discussion

In this present retrospective study, we evaluated the association between GLCM-entropy in 18F-FDG PET/CT and response to anti-PD-1/PD-L1 monotherapy at first evaluation in extensive-stage NSCLC. No significant association was found. GLCM-entropy tended to show an association between patients with PD versus non-PD patients (*p* = 0.113). Furthermore, GLCM-entropy was not significantly associated with OS or PFS.

New biomarkers and prediction tools to select the ideal NSCLC candidate population that will benefit from immunotherapy are needed. Conventional parameters based on 18F-FDG PET/CT for predicting prognosis have been investigated in patients with various types of cancer. SUVmax is an easy, robust, and reproductible parameter and was reported as a potential predictive marker of response to immunotherapy in lung cancer [22,23] in a previous study; however, this has not been verified in other studies [24]. It does not seem to be associated with OS or PFS [25,26]. SUVmax does not fully reflect tumor size or heterogeneity, which justifies investigating the MTV and TLG. The MTV represents the volume of voxels with higher 18F-FDG accumulation than the cut-off value. TLG is calculated by multiplying the MTV by the mean SUV within the lesion. Among these conventional parameters, a lower MTV in the basal 18F-FDG PET/CT was associated with better disease control, and better OS and PFS in a retrospective study analyzing 92 NSCLC patients treated with nivolumab, pembrolizumab, or atezolizumab [27]. In a prospective study looking at 75 NSCLC patients treated with PD-1 inhibitors (pembrolizumab and nivolumab), the MTV was identified as a strong prognostic and predictive factor [25].

These conventional parameters reflect tumor metabolism and tumor size, but intra-tumor heterogeneity remains to be considered. A systematic lung cancer review published in 2018 (17 studies) suggests that the prognostic value of texture analysis remains unproven [28]. Recently, a few studies have shown the prognostic value of texture parameters in disease control and survival in extensive-stage NSCLC treated with immunotherapy. In a retrospective study, Polverari et al. showed that higher heterogeneity (defined by kurtosis and skewness) was significantly associated with probability of immunotherapy failure [24]. Valentinuzzi et al. studied radiomics features in stage IV NSCLC patients treated with pembrolizumab and showed that GLCM-entropy and GLRLM-SRE were able to discriminate responders from non-responders [18].

Tumor response might be linked to tumor burden rather than the intensity of 18F-FDG uptake. Huang et al. showed that the ratio between circulating reinvigorated CD8 T cells and tumor burden as assessed by CT could predict tumor response in patients treated with immunotherapy for melanoma [29]. Chardin et al. hypothesized that patients with a high tumor burden generally have a lower reinvigorated CD8 T cell count relative to the tumor burden ratio, which could explain their lower survival rates [25]. Moreover, in this prospective study, a lower OS was observed in patients treated with first line immunotherapy than in patients who had been previously treated. The authors suggest that this difference is related to the higher MTV in patients who did not receive prior treatment (chemotherapy or local treatment), suggesting that combined treatment protocols including a phase of chemotherapy and/or radiotherapy aiming to diminish MTV could be superior to immunotherapy alone in patients with a high MTV. In our study, GLCM-entropy was not predictive of OS or PFS, whether the patient was treated with anti-PD-1/PD-L1 monotherapy in the first, second, or a subsequent line.

In our study, we used images acquired by a single scanner with a single reconstruction method. We used the LIFEx freeware for radiomic feature calculation in multi-modality imaging to characterize tumor heterogeneity. Although texture analyses might be clinically useful, there are some issues regarding their reproducibility. Grootjans et al. studied the influence of respiratory motion on the quantification of texture features in patients with lung cancer and concluded that the tested textural features (entropy, high intensity emphasis, zone percentage, and dissimilarity) were robust in the respiratory motion artefacts and varying levels of image noise [30]. A repeatability analysis on a prospective multi-center cohort looking at 74 patients with stage IIIB–IV NSCLC (73 primary tumors and 32 additional lesions were analyzed) showed that entropy was one of the most repeatable features among GLCM features (with sum entropy and difference entropy), whereas most other features fell into the reliable category [17].

The calculation of texture indices involves resampling the tumor voxel intensities within the tumor. The resampling method that we used here, known as relative resampling, consists in rescaling the tumor voxel values using a fixed number of discrete values between 0 and 1 (typically 64). Another method, known as absolute resampling, was tested by Orlhac et al. to overcome the limitations of the relative resampling approach, in which the limits used for resampling were set to 0 and 20 SUV units [31]. They showed that texture indices computed using an absolute resampling method vary as a function of the tissue type and cancer sub-type more than the texture indices in the usual relative resampling method. These results indicate that resampling can be performed differently and significantly impact the values.

To limit potential selection biases among texture features, we also performed a non-supervised analysis for all features, clustering PD vs. non-PD patients into a predictive model. Interestingly, GLCM_Entropy remained the first and main factor associated with responses to immunotherapy.

For homogeneity, our study included patients treated with ICI monotherapy only. This restriction to ICI monotherapy allowed us to specifically assess the response to immunotherapy, avoiding the potential effects associated with chemotherapy.

There are some limitations to our study. First, population selection could have been affected by the retrospective design of our study and a more homogenous population may have been selected with a prospective design (e.g., a population receiving the same immunotherapy, first line treatment only, etc.). Moreover, the sample size was limited (47 patients), and analyses on a larger cohort may have shown significant differences in the results. We also checked and verified that no confounding factors were associated with GLCM-entropy. Despite no association between GLCM-entropy and prognosis factors, such as lines of previous systemic therapies or age at first immunotherapy infusion, a larger and prospective cohort would allow for us to control potential remaining biases. Another limitation could be the choice of the primary aim of our study, i.e., assessing the response to anti-PD-1/PD-L1 monotherapy at first evaluation. Indeed, immunotherapy marshals the specificity and long-term memory of the adaptive immune response to achieve durable tumor regression and its delay of action is usually longer than other therapies. In addition, we specified that when there was no pulmonary lesion, features were extracted from the extra-pulmonary target lesion with the highest SUVmax, which means that some texture parameters were not extracted from the lung. Furthermore, we chose RECIST to evaluate response to immunotherapy; however, it does not capture the atypical patterns of tumor responses described with ICIs, which are better assessed using iRECIST, although this requires a new evaluation at 4 weeks in the case of PR, CR, or PD. Mulkey et al. compared these two criteria in patients treated with ICIs and showed that patients with initial progressive disease as per RECIST V.1.1 can experience prolonged stability or substantial reductions in tumor burden as per iRECIST [32]. However, iRECIST was not feasible in our retrospective study. Finally, re-evaluation modalities were different between the patients (PET/CT, CT, or clinical/biological by default) due to the retrospective design of our study.

## 5. Conclusions

According to our results and considering the limitations highlighted above, GLCM-entropy measured in 18F-FDG PET/CT performed before the start of ICI monotherapy in extensive-stage NSCLC does not appear to be predictive of response at first follow-up.

In order to select the population that may benefit from immunotherapy, novel prediction tools are needed. To this end, radiomics offers the advantage of being non-invasive and widely available. This study demonstrates the feasibility of entropy assessments in 18F-FDG PET/CT realized in routine clinical practice. Despite the negative results of our retrospective study regarding the prediction of response to ICI monotherapy, we believe that new PET parameters that could be associated with other clinical/biological parameters should be tested in larger prospective studies in order to provide better selection criteria to predict response to ICI therapy.

## Figures and Tables

**Table 1:** Patient characteristics.

Characteristics	Value
Age	
Median (range)-year	64 (32–84)
Distribution-n (%)	
<65 year	25 (53.2)
≥65 year	22 (46.8)
Sex-n (%)	
Male	33 (70.2)
Female	14 (29.8)
ECOG ^1^ performance status score-n (%)	
0	16 (34.0)
1	21 (44.7)
2	9 (19.2)
3	1 (2.1)
Histologic type of tumor-n (%)	
Adenocarcinoma	30 (63.8)
Squamous cell carcinoma	15 (31.9)
Other (poorly differentiated, not otherwise specified)	2 (4.3)
Smoking status-n (%)	
Never smoked	6 (12.8)
Current or former smoker	40 (85.1)
Unknown	1 (2.1)
PD-L1 expression level-n (%)	
<1%	11 (23.4)
1–49%	7 (14.9)
≥50%	15 (31.9)
Unknown	14 (29.8)
Immunotherapy-n (%)	
Atezolizumab	2 (4.3)
Nivolumab	25 (53.2)
Pembrolizumab	20 (42.5)
Lines of previous systemic therapy-n (%)	
0	15 (31.9)
1	19 (40.4)
≥ 2	13 (27.7)

^1^ ECOG: Eastern Cooperative Oncology Group performance status scores range from 0 to 5, with higher scores indicating greater disability.

**Table 2:** PET parameters.

PET Parameters	Minimum	Median	Maximum
SUVmax (g/mL)	2.07	10.92	37.03
SUVmean (g/mL)	0.64	2.77	5.97
TLG (g)	4.12	142.36	4053.43
MTV (mL)	2.14	54.07	1696.19
GLCM-entropy	2.30	5.78	7.81
GLRLM-SRE	0.53	0.81	0.92

**Table 3:** Association between main PET parameters and PD vs. non-PD.

PET Parameters	Status	Median	*p* Value
SUVmax (g/mL)	PD	8.02	0.103
	Non-PD	13.04	
SUVmean (g/mL)	PD	2.54	0.519
	Non-PD	2.89	
TLG (g)	PD	192.54	0.428
	Non-PD	105.28	
MTV (mL)	PD	57.63	0.346
	Non-PD	30.87	
GLCM-entropy	PD	5.56	0.113
	Non-PD	6.15	
GLRLM-SRE	PD	0.80	0.727
	Non-PD	0.81	

**Table 4:** Comparisons of patient characteristics according to GLCM-entropy.

Characteristics	Value		*p*-Value
	**GLCM-Entropy**		
	<median (n = 24)	≥median (n = 23)	
Age			
Median (range)-year	64 (44–84)	64 (32–82)	0.975
Sex-n (%)			
Male	17 (70.8)	16 (69.6)	0.924
Female	7 (29.2)	7 (30.4)	
ECOG ^1^ performance status score-n (%)			
0	8 (33.3)	8 (34.8)	0.677
1	10 (41.7)	11 47.8)	
2	5 (20.8)	4 17.4)	
3	1 (4.2)	0 (0)	
Histologic type of tumor-n (%)			
Adenocarcinoma	17 (70.8)	13 (63.8)	0.572
Squamous cell carcinoma	6 (25.0)	9 (31.9)	
Other (poorly differentiated, not otherwise specified)	1 (4.2)	1 (4.3)	
Smoking status-n (%)			
Never smoked	2 (8.3)	4 (17.4))	0.472
Current or former smoker	22 (91.7)	18 (78.3)	
Unknown	0 (0)	1 (4.3)	
PD-L1 expression level-n (%)			
<1%	6 (25.0)	5 (21.7)	0.572
1–49%	5 (20.8)	2 (8.7)	
≥50%	6 (25.0)	9 (39.1)	
Unknown	7 (29.2)	7 (30.4)	
Immunotherapy-n (%)			
Atezolizumab	1 (4.2)	1 (4.3)	0.763
Nivolumab	14 (58.3)	11 (47.8)	
Pembrolizumab	9 (37.5)	11 (47.8)	
Lines of previous systemic therapy-n (%)			
0	7 (29.2)	8 (34.8)	0.114
1	13 (54.2)	6 (26.1)	
≥2	4 (16.7)	9 (39.1)	

^1^ ECOG: Eastern Cooperative Oncology Group performance status scores range from 0 to 5, with higher scores indicating greater disability.

**Table 5:** Multi-variate analysis factors associated with progressive disease vs. non-progressive disease for patients treated with immunotherapy in global population.

Factor	HR (95% CI)	*p*-Value
Age	0.94 [0.87–1.02]	0.15
Male sex	0.55 [0.04–6.99]	0.65
Current smokers	1 [1–1]	1
Sub-type histology	1.05 [0.13–8.25]	0.96
ECOG PS-0	0.42 [0.02–6.9]	0.548
No previous treatment	6.05 [0.6–61.07]	0.13
PDL-1 > 50%	0.09 [0–11.31]	0.33
GLCM-entropy < median	0.14 [0.02–0.79]	0.03

## Data Availability

The data presented in this study are available on request from the corresponding author.

## Supplementary Materials

The following supporting information can be downloaded at: https://www.mdpi.com/article/10.3390/life13041051/s1, Figure S1: Association between GLCM-entropy and OS for patients treated in the second line or subsequent treatments. Kaplan–Meier plot showing prognostic value of GLCM-entropy for the overall survival (OS) (*p* = 0.068). Crosses represent censored patients; Table S1: Correlation matrix between each simple and complexe texture parameters.
