# Supplementary material for: Assessment of the Association between Entropy in PET/CT and Response to Anti-PD-1/PD-L1 Monotherapy in Stage III or IV NSCLC
Source: Life (Basel). 2023 Apr 20;13(4):1051. doi: 10.3390/life13041051 (PMC10142835; doi:10.3390/life13041051)
Supplement: Supplementary file 1 [file life-13-01051-s001.zip › life-2303899-supplementary Figure S1.pdf]

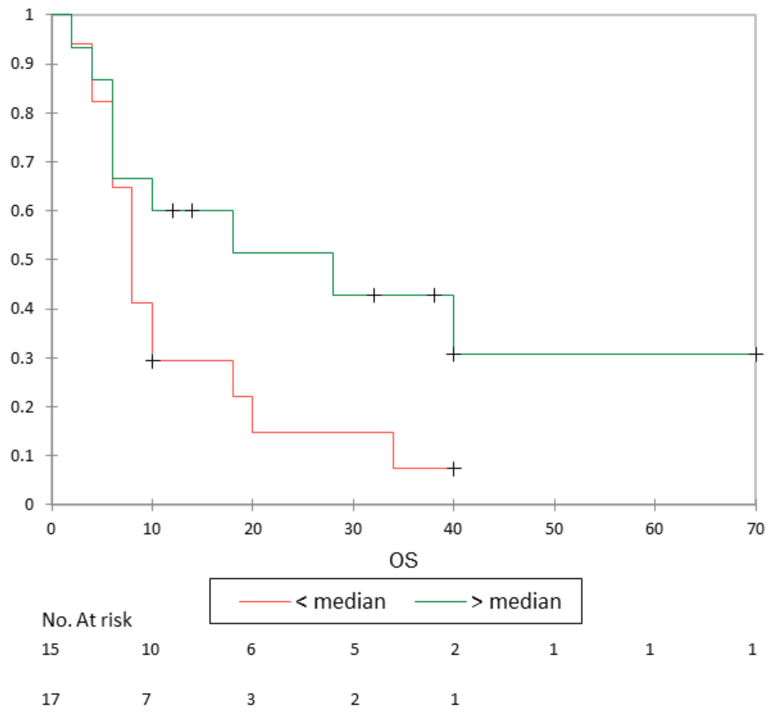

Figure S1. Association between GLCM-entropy and OS for patients treated in second line or more. Kaplan–Meier plot showing prognostic value of GLCM-entropy on the overall survival (OS) (p = 0.068). Crosses represent censored patients.
